# Micro-computed tomography assessment of bone structure in aging mice

**DOI:** 10.1038/s41598-022-11965-4

**Published:** 2022-05-17

**Authors:** Junbo Shim, Chihiro Iwaya, Catherine G. Ambrose, Akiko Suzuki, Junichi Iwata

**Affiliations:** 1 Department of Diagnostic and Biomedical Sciences, School of Dentistry, The University of Texas Health Science Center at Houston, 1941 East Road, BBS 4208, Houston, TX 77054 USA; 2 Center for Craniofacial Research, School of Dentistry, The University of Texas Health Science Center at Houston, Houston, TX 77054 USA; 3 Department of Orthopedic Surgery, McGovern Medical School, The University of Texas Health Science Center at Houston, Houston, TX 77030 USA; 4 Pediatric Research Center, McGovern Medical School, The University of Texas Health Science Center at Houston, Houston, TX 77030 USA; 5 MD Anderson UTHealth Graduate School of Biomedical Sciences, Houston, TX 77030 USA

**Keywords:** Bone, Ageing, Bone

## Abstract

High-resolution computed tomography (CT) is widely used to assess bone structure under physiological and pathological conditions. Although the analytic protocols and parameters for micro-CT (μCT) analyses in mice are standardized for long bones, vertebrae, and the palms in aging mice, they have not yet been established for craniofacial bones. In this study, we conducted a morphometric assessment of craniofacial bones, in comparison with long bones, in aging mice. Although age-related changes were observed in the microarchitecture of the femur, tibia, vertebra, and basisphenoid bone, and were more pronounced in females than in males, the microarchitecture of both the interparietal bone and body of the mandible, which develop by intramembranous ossification, was less affected by age and sex. By contrast, the condyle of the mandible was more affected by aging in males compared to females. Taken together, our results indicate that mouse craniofacial bones are uniquely affected by age and sex.

## Introduction

The skeleton performs a variety of functions such as providing structural support, enabling movement, protecting vital internal organs, and maintaining mineral homeostasis, acid–base balance, and hematopoiesis^1^. There are two major types of bone: cortical and trabecular bone. Cortical bone consists of lamellar units called osteons and forms a solid osseous shell around the bone; the outer surface of cortical bone is covered by the periosteum, whereas the inner surface is covered by the endosteum. Trabecular bone is composed of a network of lamellar bone plates and rods and is deposited by diffusion from the surrounding bone marrow^1^.

Bone ossification starts at the embryo stage and continues until approximately age 25 years in humans^2^. Bone ossification is achieved through intramembranous and endochondral ossification. Whereas intramembranous ossification involves the direct conversion of the mesenchyme to bone and forms the flat bones of the skull, clavicle, and most of the cranial bones, endochondral ossification involves a cartilage template that is later replaced by bone and forms the long bones and cranial base^2^. Bones are continuously and actively remodeled throughout life; therefore, bone morphology and matrix quality change during development and with aging.

Disruption of the homeostasis between bone formation by osteoblasts and bone resorption by osteoclasts results in imbalanced bone remodeling. For instance, osteopenia, a condition of low bone mass or low bone density, occurs when bone resorption exceeds bone formation, and its progression is influenced by diet, environmental factors, hormonal changes, and aging. Individuals with osteopenia have a higher risk of suffering bone fractures and developing osteoporosis, in which bone mineral density (BMD) measured by dual X-ray absorptiometry, the gold standard for measuring age-related changes, is significantly reduced by more than two-fold^3–5^.

In osteoporotic bones, bone remodeling shifts toward bone resorption, resulting in increased net bone loss with aging^6^. In addition, this bone loss depends on the amount of surface available for bone remodeling^7^. Therefore, bone loss in early osteoporosis is mainly a loss of trabecular bone due to the larger surface available for bone remodeling^8^. However, cortical bone becomes more porous with aging, which leads to increased endocortical surface. As a result, bone loss in late osteoporosis is mainly a loss of cortical bone^7^. Previous studies indicate that cortical bone strength in the femur^9,10^, as well as cortical thickness in various bones, decrease with aging^11,12^. In addition, trabecular bone loss has been observed in the femoral head and vertebrae with aging, along with decreased trabecular bone volume and increased trabecular bone separation^11,13,14^.

Primary osteoporosis is associated with menopause in women (type I) and normal aging (type II), whereas secondary osteoporosis is caused by certain medical conditions such as inflammatory disease, type II diabetes, and cancer, and risk factors including corticosteroid medication and high alcohol consumption^15–17^. Type I postmenopausal osteoporosis is characterized by high bone turnover with increased osteoblastic and osteoclastic activities and rapid trabecular bone loss (3–8 times higher)^18,19^ and develops between 50 and 70 years of age. In type II senile osteoporosis, there is low bone turnover, with reduction of both osteoblastic and osteoclastic activities, and gradual loss of trabecular and cortical bone (two-fold higher in women)^6,20^.

Although both estrogen and androgen inhibit bone resorption and enhance bone formation, estrogen plays a more dominant role in the inhibition of bone resorption^21^. This is evident in women with estrogen deficiency due to menopause, which is associated with rapid trabecular bone loss^6^. The prevalence of osteoporosis is, therefore, higher in postmenopausal women than in older men. The National Osteoporosis Foundation has estimated that 9.1 million women are affected by osteoporosis, while only 2.8 million men develop the disease. Nonetheless, older men still suffer poor health outcomes resulting from osteoporosis.

Long bones and the vertebrae have been rigorously studied in both human and mice, as they are common sites of fracture due to aging and osteopenia/osteoporosis. By contrast, traumatic injuries and fractures in craniofacial bones are most commonly caused by falls and motor vehicle accidents^22,23^. Although recent efforts have been made to quantify the bone morphometry of the skull (e.g. cortical thickness, density, and porosity), the trabecular morphometry of the diploe has not been studied in detail^24–26^.

Micro-computed tomography (μCT) allows for the visualization of three-dimensional (3D) structures and is widely used to evaluate bone quality and morphology in small animals, under physiological and pathological conditions. μCT analyses are useful for evaluating the fine structure of bone/cartilage mineral components, particularly in the field of bone and cartilage research^27–30^. Qualifiable and quantifiable analyses of long bones and vertebrae, with a set of standardized parameters, are well established in the field; by contrast, a standardized protocol for craniofacial bones has not been yet defined. While some craniofacial bones are formed through endochondral ossification (e.g. the mandibular condyle and cranial base^31,32^), most are formed through intramembranous ossification. A few μCT studies have been conducted in young mice^33^, but a rigorous methodology for evaluating aging craniofacial bones has not been tested and validated. This study thus aims to evaluate the changes that occur in the structural properties of craniofacial bones in aging mice through μCT imaging analyses.

## Materials and methods

### Animals

Male and female C57BL/6J mice were purchased from The Jackson Laboratory at 26 weeks (6 months), 56 weeks (12 months), 72 weeks (18 months), and 90 weeks (22 months) of age (n = 3 per group), and housed with a 12-h light/12-h dark cycle and fed a normal mouse diet ad libitum until μCT scanning. Mice were euthanized through carbon dioxide overdose followed by cervical dislocation at the time points indicated above. The femur, tibia, vertebrae, and the skull were dissected and fixed with 4% paraformaldehyde (PFA) overnight, and then stored in phosphate-buffered saline (PBS) for subsequent scanning. The surrounding connective tissues and muscles were removed before the scan. The protocol was approved by the Animal Welfare Committee (AWC) and the Institutional Animal Care and Use Committee (IACUC) of UTHealth (AWC 19-0079). All mice were maintained at the animal facility of UTHealth, and all animal experiments were conducted according to the Animal Research: Reporting of in vivo experiments (ARRIVE) guidelines on ethical conduct in animal research.

### μCT scanning and 3D reconstruction

Samples were placed in a foam mold for stability during the μCT scans, which were performed at 15 µm resolution for the head, third lumbar vertebra, tibia, and femur using a SCANCO vivaCT-40 system (SCANCO Medical AG, Fabrikweg, Switzerland; 70 peak kilovoltage and 145-µA X-ray source). Air was used as the scan medium to provide the highest contrast between the sample and the background. 3D reconstruction and analysis of the μCT images were performed with the Dragonfly software [Version 2021.1 for Windows; Object Research Systems (ORS) Inc., Montreal, Canada] with DICOM files.

### Determination of volume of interest

The difference in bone size according to age was taken into consideration when determining the volume of interest (VOI) for all bones. For the femur, trabecular bone measurements were taken 0.5 mm proximally from the distal epiphyseal growth plate, with 1 mm in height, and cortical bone parameters were measured at mid-diaphysis (which was located by taking the midpoint between the greater trochanter and intercondylar notch), extending to a length of 0.5 mm. For the tibia, trabecular bone measurements were taken 0.5 mm distally from the tibial metaphysis, with 1 mm in height, and cortical bone parameters were measured at the midpoint between the distal and proximal growth plates, extending to a length of 0.5 mm. For the vertebrae, the VOI for the body of the third lumbar vertebrae (L3) was determined relative to the height of the vertebra; measurements were taken from a VOI of 50% in height and 45% in width from the center of the body, and cortical thickness was calculated from the ventral vertebral cortex.

For craniofacial bones, several candidate bones were selected based on the clinical relevance and clear/ rich trabecular architecture. For the basisphenoid bone, VOI was taken from 200 µm anteriorly to the spheno-occipital synchondrosis (SOS), with 1 mm in width and 500 µm in length. For the interparietal bones, VOI was taken from the largest cross-sectional area, with 1.5 mm in width and 1 mm in length. For the mandibular trabecular bone, VOI was located at the first molar (M1) bifurcation, extending 30 slices between the M1 proximal and distal roots; trabecular bone below the M1 roots was outlined excluding the molars, lower incisors, and the mandibular cortical bone. For the mandibular condyle, the most anterior and posterior points of the mandibular condyle were located, and the VOI of the condyle was selected above the artificial line drawn from the anterior to the posterior points.

### Imaging analysis

The Otsu’s thresholding method was used to segment bone from non-bone. The Bone Analysis module in Dragonfly was used to segment cortical and trabecular bone^34,35^ and to compute bone morphometric measurements, following the guidelines described by Bouxsein et al.^28^. Using the Bone Analysis module, trabecular bone volume fraction (BV/TV), trabecular thickness (Tb.Th, µm), and trabecular separation (Tb.Sp, µm) were determined for trabecular bone; trabecular number (Tb.N, 1/ mm) was calculated based on 3D measurements for the spacing of trabeculae^36,37^ using the following formula:

$$Tb.N=\frac{1}{(Tb.Th+Tb.Sp)}$$

Cortical area (Ct.Ar, µm^2^), total area (Tt.Ar, µm^2^), cortical area fraction (Ct.Ar/Tt.Ar, %), and cortical thickness (Ct.Th, µm) were determined for cortical bone.

### Mineral density measurements

Mineral density measurements were captured with the Scanco software. Calibration was performed weekly, using a bone calibration phantom with one of five densities of hydroxyapatite (HA; 0, 100, 200, 400, 800 mg HA/cm^3^). For trabecular bone, density was reported as BMD in mg HA/cm^3^; for cortical bone, density was reported as tissue mineral density (TMD) in mg HA/cm^3^. Following segmentation, the following settings were applied to all mineral density measurements: Gauss Sigma = 1, Gauss Support = 0, Lower Threshold = 210, and Upper Threshold = 1000 (Threshold ranging from 0 to 1000).

### Statistical analysis

All experimental data were analyzed with the Prism software (GraphPad Software, California, USA). One-way or two-way analysis of variance (ANOVA) with Bonferroni correction was used for the analyses (n = 3). A *p*-value < 0.05 was considered statistically significant. For all graphs, data are represented as mean ± standard deviation (SD).

## Results

### Aged-related changes in the femur, tibia, and vertebrae

**Figure 1:**
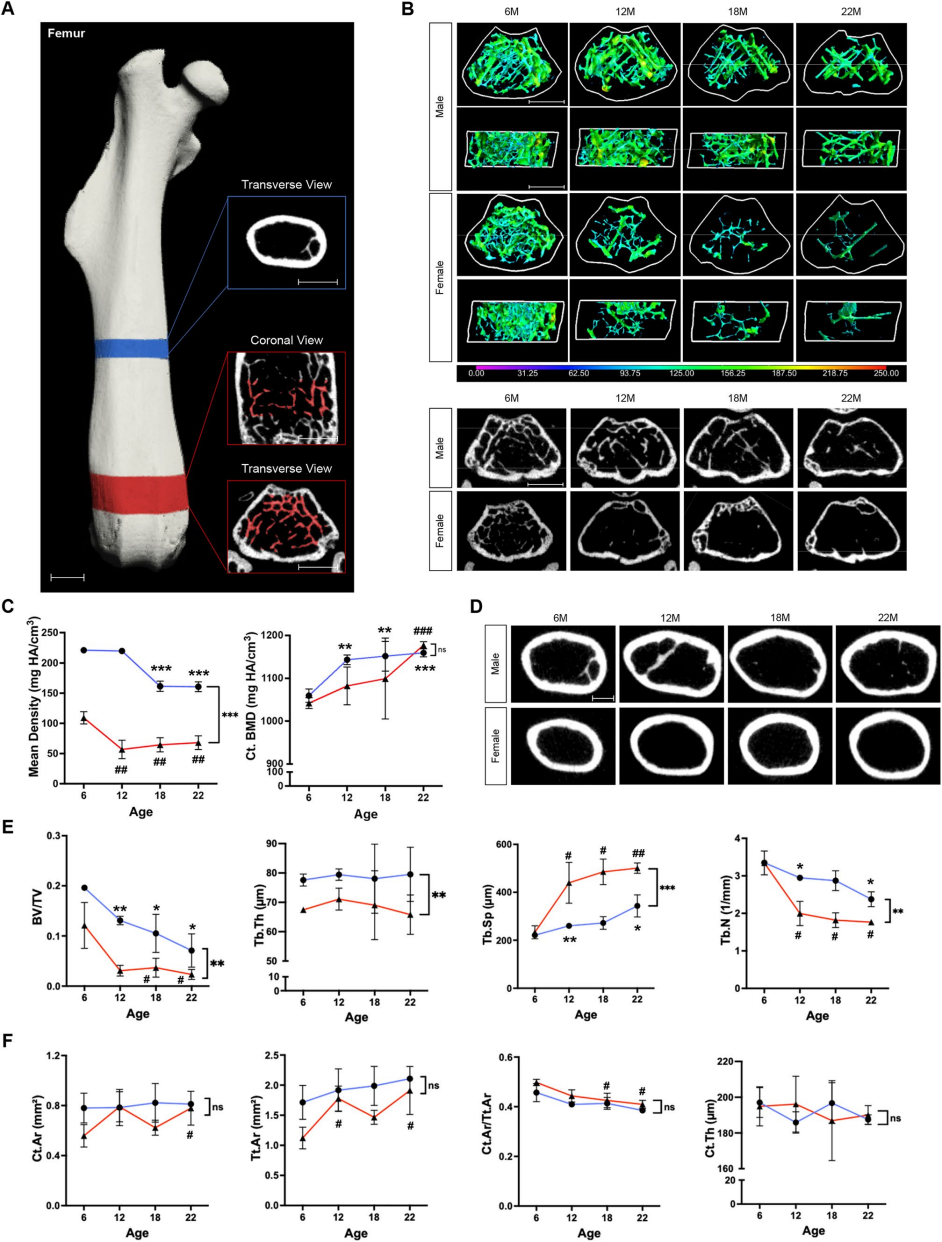
μCT imaging analysis of the femur. (**A**) Overview of VOI selection for the femur. VOI for cortical bone at mid-diaphysis is shown in the blue panel, VOI for trabecular bone at distal metaphysis is shown in the red panels. Scale bar, 1 mm. (**B**) Colored 3D reconstruction (top) and 2D cross-sectional images (bottom) of trabecular bone in the femur of male and female mice at 6, 12, 18, and 22 months of age. Color bar shows trabecular bone thickness from 0 to 250 µm. Scale bar, 1 mm. (**C**) Femoral trabecular bone mineral density as Tb.BMD in mg HA/cm^3^ and cortical bone mineral density as Ct.TMD in mg HA/cm^3^ for male (blue lines) and female (red lines) mice at 6, 12, 18, 22 months of age. (**D**) Original transverse cross-sectional images of cortical bone in the femur of male and female mice at 6, 12, 18, and 22 months of age. Scale bar, 1 mm. (**E**) Quantification of morphometric parameters of the femoral trabecular bone of males (blue lines) and females (red lines): trabecular bone volume fraction (BV/TV), trabecular number (Tb.N, 1/mm), trabecular thickness (Tb,Th, µm), and trabecular separation (Tb.Sp, µm). (**F**) Quantification of morphometric parameters of femoral cortical bone of males (blue lines) and females (red lines). Cortical area (Ct.Ar, mm^2^), total area (Tt.Ar, mm^2^), cortical area fraction (Ct.Ar/Tt.Ar), and cortical thickness (Ct.Th, µm). n = 3 per group. *p < 0.05, **p < 0.01, ***p < 0.001 for males; ^#^*p* < 0.05, ^##^*p* < 0.01, ^###^*p* < 0.001 for females; ns, not significant.

In an effort to compare our methodology with that used in previous studies, we evaluated the structure of the femur, tibia, and vertebra. Previous studies have shown that age-related changes in these bones lead to a decrease in trabecular bone volume fraction and an increase in trabecular separation^38,39^. To evaluate the age-related changes in the femur, we analyzed trabecular bone (Fig. 1A) and found that femoral BV/TV in male and female mice decreased by 63.8% (*p* < 0.05) and 80.8% (*p* < 0.05), respectively, from 6 to 22 months of age. In addition, there was a significant difference in BV/TV between the sexes over time (*p* < 0.01). In correlation with a decreased BV/TV, there was a significant increase in Tb.Sp—88.3% (*p* < 0.05) in females and 17.7% (*p* < 0.01) in males—and a significant decrease in Tb.N—40.3% (*p* < 0.05) in females and 12.1% (*p* < 0.05) in males—from 6 to 12 months of age. Tb.BMD decreased by 27.4% (*p* < 0.001) in males, and by 41% (*p* < 0.001) in females, from 6 to 22 months. By contrast, femoral Tb.Th did not significantly change throughout life in both males and females. As expected, sex differences were observed for Tb.Th, Tb.Sp, Tb.N, and Tb.BMD (Fig. 1B,C,E).

Femoral Ct.Ar did not change from 6 to 22 months of age in both male and female mice, while there was a gradual increase in Tt.Ar in both sexes. In correlation with increased Tt.Ar, there was a slight reduction in Ct.Ar/Tt.Ar in females (17.8%; *p* < 0.05) over time. Ct.TMD increased by 9.6% (*p* < 0.001) in males and by 11.8% (*p* < 0.001) in females. Ct.Th did not change significantly throughout life (Fig. 1C,D,F).

**Figure 2:**
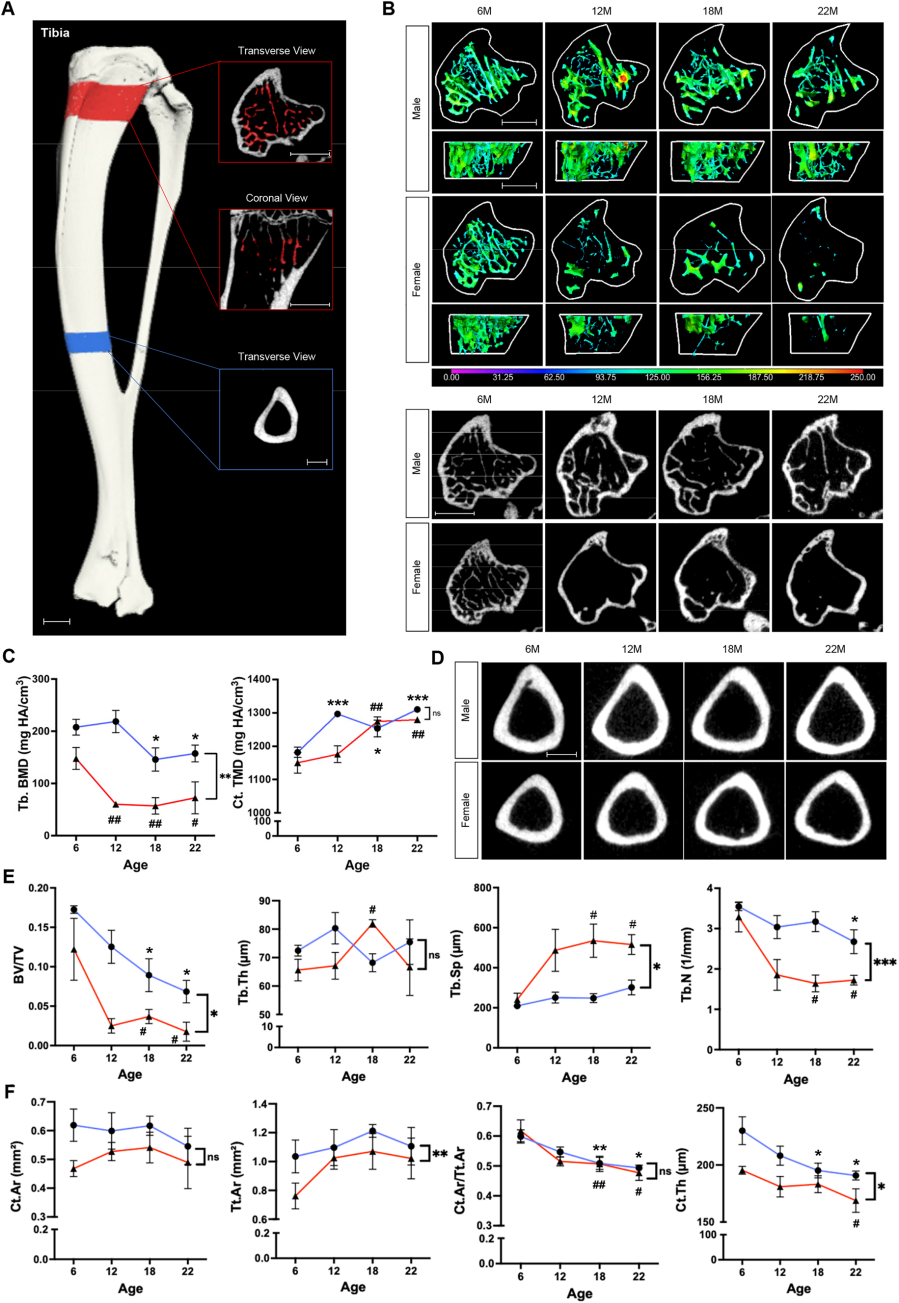
μCT imaging analysis of the tibia. (**A**) Overview of VOI selection for the tibia. VOI for cortical bone at mid-diaphysis is shown in the blue panel, VOI for trabecular bone at proximal metaphysis is shown in the red panels. Scale bar, 1 mm. (**B**) Colored 3D reconstruction (top) and 2D cross-sectional images (bottom) of trabecular bone in the tibia of male and female mice at 6, 12, 18, and 22 months of age. Color bar shows trabecular bone thickness from 0 to 250 µm. Scale bar, 1 mm. (**C**) Tibial trabecular bone mineral density as Tb.BMD in mg HA/cm^3^ and cortical bone mineral density as Ct.TMD in mg HA/cm^3^ for male (blue lines) and female (red lines) mice at 6, 12, 18, 22 months of age. (**D**) Original transverse cross-sectional images of cortical bone in the tibia of male and female mice at 6, 12, 18, and 22 months of age. Scale bar, 1 mm. (**E**) Quantification of morphometric parameters of tibial trabecular bone of males (blue lines) and females (red lines). Trabecular bone volume fraction (BV/TV), trabecular number (Tb.N, 1/mm), trabecular thickness (Tb,Th, µm), and trabecular separation (Tb.Sp, µm). (**F**) Quantification of morphometric parameters of tibial cortical bone of males (blue lines) and females (red lines). Cortical area (Ct.Ar, mm^2^), total area (Tt.Ar, mm^2^), cortical area fraction (Ct.Ar/Tt.Ar), and cortical thickness (Ct.Th, µm). n = 3 per group. *p < 0.05, **p < 0.01, ***p < 0.001 for males; ^#^*p* < 0.05, ^##^*p* < 0.01, ^###^*p* < 0.001 for females; ns, not significant.

We found that trabecular bone morphometrics of the tibia (Fig. 2A) followed similar patterns with aging: BV/TV decreased by 60.4% (*p* < 0.05) in males and by 85.6% (*p* < 0.05) in females; Tb.N decreased by 24.6% (*p* < 0.05) in males and by 47.6% (*p* < 0.05) in females; Tb.Sp increased by 43.9% (*p* < 0.05) in males and by 114.0% (*p* < 0.05) in females; and Tb.BMD decreased by 24.3% (*p* < 0.05) in males and by 51% (*p* < 0.05) in females over time. In female mice, substantial changes in these measurements were observed from 6 to 12 months of age. Sex differences were observed for Tb.Th, Tb.Sp, Tb.N, and Tb.BMD (Fig. 2B,C,E).

Cortical bone parameters for the tibia showed patterns similar to those seen in the femur, with slight reduction in Ct.Ar/Tt.Ar in both males (17.6%; *p* < 0.05) and females (22.7%; *p* < 0.05) over time. Tibial Ct.Th significantly decreased throughout life in both male (17.1%; *p* < 0.05) and female mice (13.6%; *p* < 0.05). Ct.TMD increased by 10.9% (*p* < 0.001) in males and by 11.2% (*p* < 0.001) in females over time. There was a difference between the sexes for Tt.Ar and Ct.Th, but not for Ct.Ar, CtAr/Tt.Ar and Ct.TMD (Fig. 2C,D,F).

**Figure 3:**
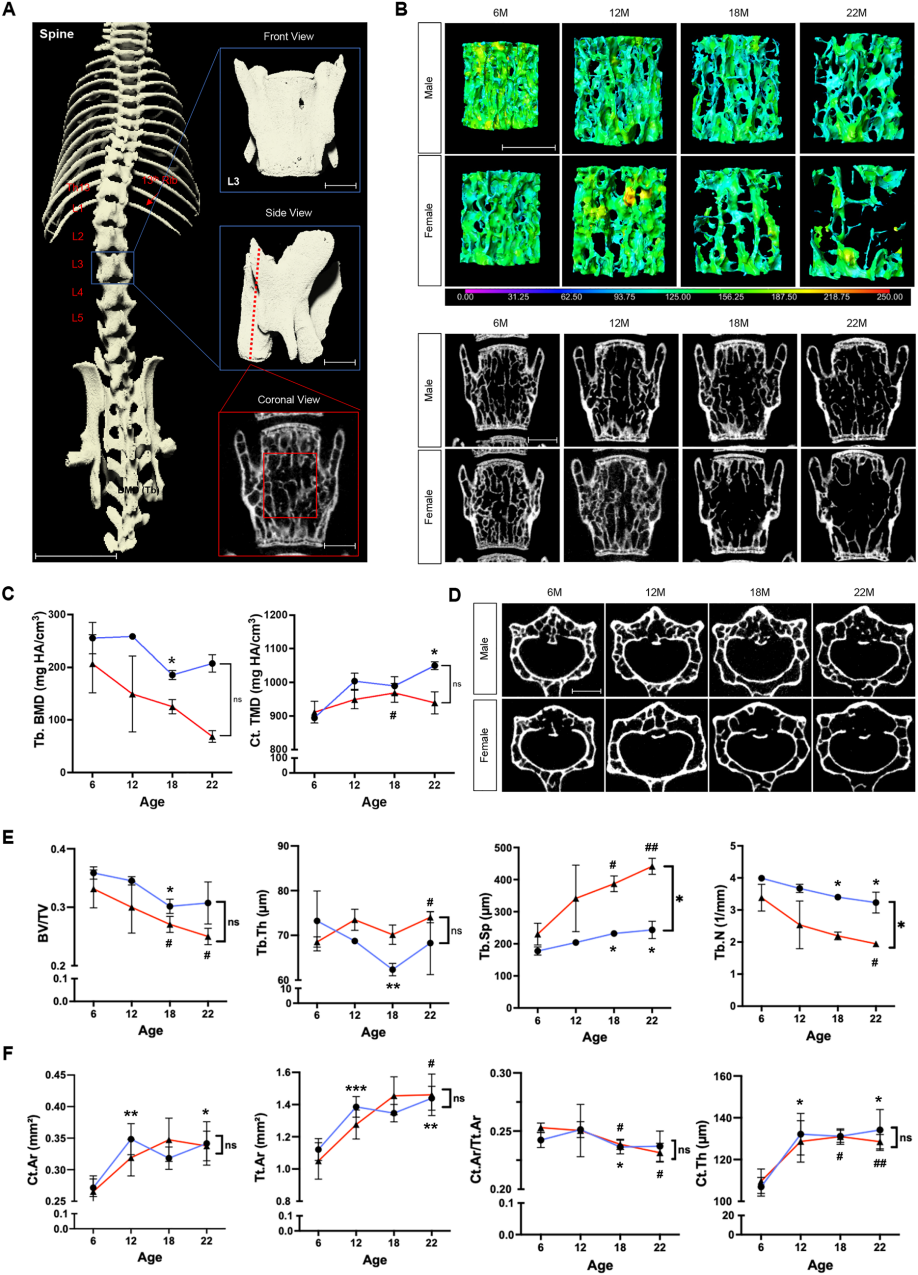
μCT imaging analysis of the third lumbar vertebra. (**A**) Overview of VOI selection for the third lumber vertebra (L3). Scale bar for the spine, 1 cm. Scale bar for L3, 1 mm. Th13: 13th thoracic vertebra. (**B**) Colored 3D reconstruction (top) and 2D cross-sectional images (bottom) of trabecular bone in the L3 vertebrae of male and female mice at 6, 12, 18, and 22 months of age. Color bar shows trabecular bone thickness from 0 µm to 250 µm. Scale bar, 1 mm. (**C**) Vertebral trabecular bone mineral density as Tb.BMD in mg HA/cm^3^ and cortical bone mineral density as Ct.TMD in mg HA/cm^3^ for male (blue lines) and female (red lines) mice at 6, 12, 18, 22 months of age. (**D**) Original transverse cross-sectional images of cortical bone in L3 of male and female mice at 6, 12, 18, and 22 months of age. Scale bar, 1 mm. (**E**) Quantification of morphometric parameters of L3 vertebral trabecular bone of males (blue lines) and females (red lines). Trabecular bone volume fraction (BV/TV), trabecular number (Tb.N, 1/mm), trabecular thickness (Tb,Th, µm), and trabecular separation (Tb.Sp, µm). (**F**) Quantification of morphometric parameters of L3 vertebral cortical bone of males (blue lines) and females (red lines). Cortical area (Ct.Ar, mm^2^), total area (Tt.Ar, mm^2^), cortical area fraction (Ct.Ar/Tt.Ar), and cortical thickness (Ct.Th, µm). n = 3 per group. *p < 0.05, **p < 0.01, ***p < 0.001 for males; ^#^*p* < 0.05, ^##^*p* < 0.01, ^###^*p* < 0.001 for females; ns, not significant.

In the vertebrae, a VOI (relative to the height of the vertebra) of 50% in height and 45% in width from the center of the vertebral body of L3 was analyzed for trabecular bone measurements. In addition, the ventral vertebral cortex was analyzed for cortical thickness (Fig. 3A). The trabecular bone morphometrics of the spine showed aging patterns that were similar to those seen in the femur and tibia: BV/TV decreased by 41.1% (*p* < 0.05) in females; Tb.N decreased by 19.1% (*p* < 0.05) in males and by 42.5% (*p* < 0.05) in females; Tb.Sp increased by 37.2% (*p* < 0.05) in males and by 91.8% (*p* < 0.01) in females; and Tb.TMD decreased by 66.9% (*p* < 0.05) in females over the animals’ lifespan. There was no significant change in Tb.Th with aging, but there was a difference between the sexes in Tb.Sp and Tb.N, although not in BV/TV, Tb.Th, and Tb.BMD (Fig. 3B,C,E).

Interestingly, the spine cortical morphometrics were different from those of the femur and tibia. Ct.Ar increased by 25.9% (*p* < 0.05) in males, Tt.Ar increased by 28.4% (*p* < 0.01) in males and by 39.2% (*p* < 0.05) in females, Ct.Ar/Tt.Ar decreased by 8.5% (*p* < 0.05) in females, Ct.Th increased by 25.5% (*p* < 0.05) in males and by 17.3% (*p* < 0.01) in females, and Ct.TMD increased by 17.4% (*p* < 0.05) in males over time. There was no difference in Ct.Ar, Tt.Ar, Ct.Ar/Tt.Ar, Ct.Th, and Ct.TMD between the sexes (Fig. 3C,D,F).

Our observations of age-related changes in the trabecular bone of the femur, tibia, and lumbar vertebra were consistent with previous findings, showing an increase in trabecular separation and decrease in trabecular BV/TV with aging in both male and female mice. For femoral and tibial Tb.Th measurements, there was an initial increase and a subsequent decrease as mice approached the end of their lives. Femoral and tibial cortical thickness decreased slightly, whereas vertebral cortical thickness increased slightly with aging, a similar pattern observed by Glatt et al.^39^ and Halloran et al.^40^. Cortical and trabecular measurements for female mice showed a higher degree and faster rate of bone loss compared to male mice.

### Age-related changes in craniofacial bones

**Figure 4:**
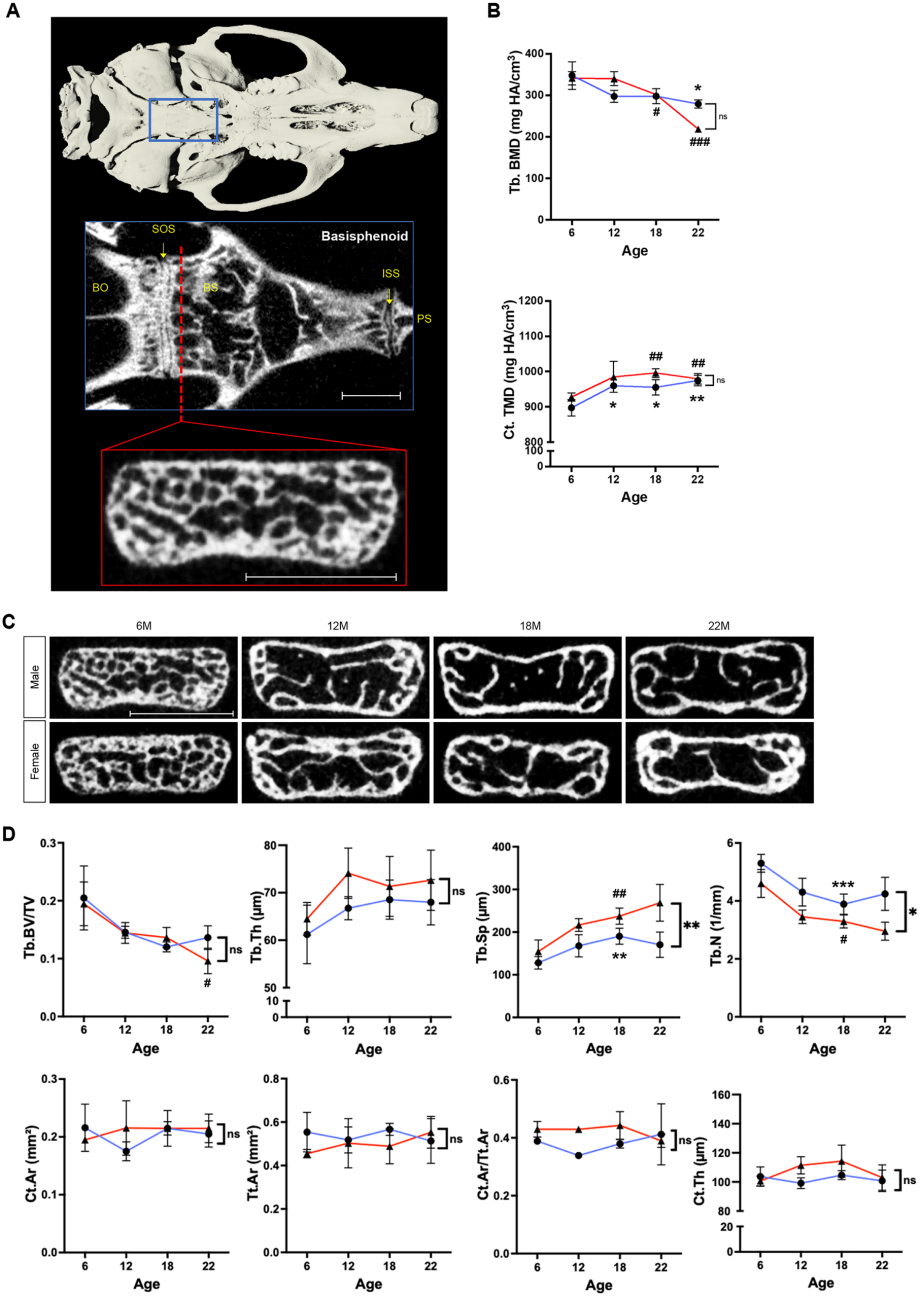
μCT imaging analysis of the basisphenoid bone. (**A**) 3D reconstruction of the skull and overview of VOI selection for the basisphenoid bone (red panel). Scale bar, 1 mm. BO, basioccipital; BS, basisphenoid; ISS, intersphenoid synchondrosis; PS, presphenoid; SOS, spheno-occipital synchondrosis. (**B**) Basisphenoid trabecular bone mineral density as Tb.BMD in mg HA/cm^3^ and cortical bone mineral density as Ct.TMD in mg HA/cm^3^ for male (blue lines) and female (red lines) mice at 6, 12, 18, 22 months of age. (**C**) Original coronal cross-sectional images of the basisphenoid bone of male and female mice at 6, 12, 18, and 22 months of age. Scale bar, 1 mm. (**D**) Quantification of morphometric parameters of the basisphenoid bone of males (blue lines) and females (red lines). Trabecular bone volume fraction (BV/TV), trabecular number (Tb.N, 1/mm), trabecular thickness (Tb,Th, µm), trabecular separation (Tb.Sp, µm), cortical area (Ct.Ar, mm^2^), total area (Tt.Ar, mm^2^), cortical area fraction (Ct.Ar/Tt.Ar), and cortical thickness (Ct.Th, µm). n = 3 per group. *p < 0.05, **p < 0.01, ***p < 0.001 for males; ^#^*p* < 0.05, ^##^*p* < 0.01, ^###^*p* < 0.001 for females; ns, not significant.

The basisphenoid bone at the cranial base is formed through endochondral ossification^41,42^, which is similar to that occurring in the long bones (Fig. 4A). From 6 to 22 months of age, BV/TV decreased by 50.6% (*p* < 0.05) in females, and Tb.BMD decreased by 19.7% (*p* < 0.05) in males and by 35.9% (*p* < 0.001) in females; however, other differences in trabecular morphometrics were not significant for both male and female mice. There was a difference in Tb.Sp and Tb.N between the sexes, but not in BV/TV, Tb.Th, and Tb.BMD. In cortical bone, Ct.TMD increased by 8.6% (*p* < 0.01) in males and by 5.6% (*p* < 0.01) in females over time. No significant changes were observed in cortical bone morphometrics for both male and female mice (Fig. 4B,C,D). Taken together, although the mode of ossification is similar, the basisphenoid bone was less affected by aging compared to the long bones.

**Figure 5:**
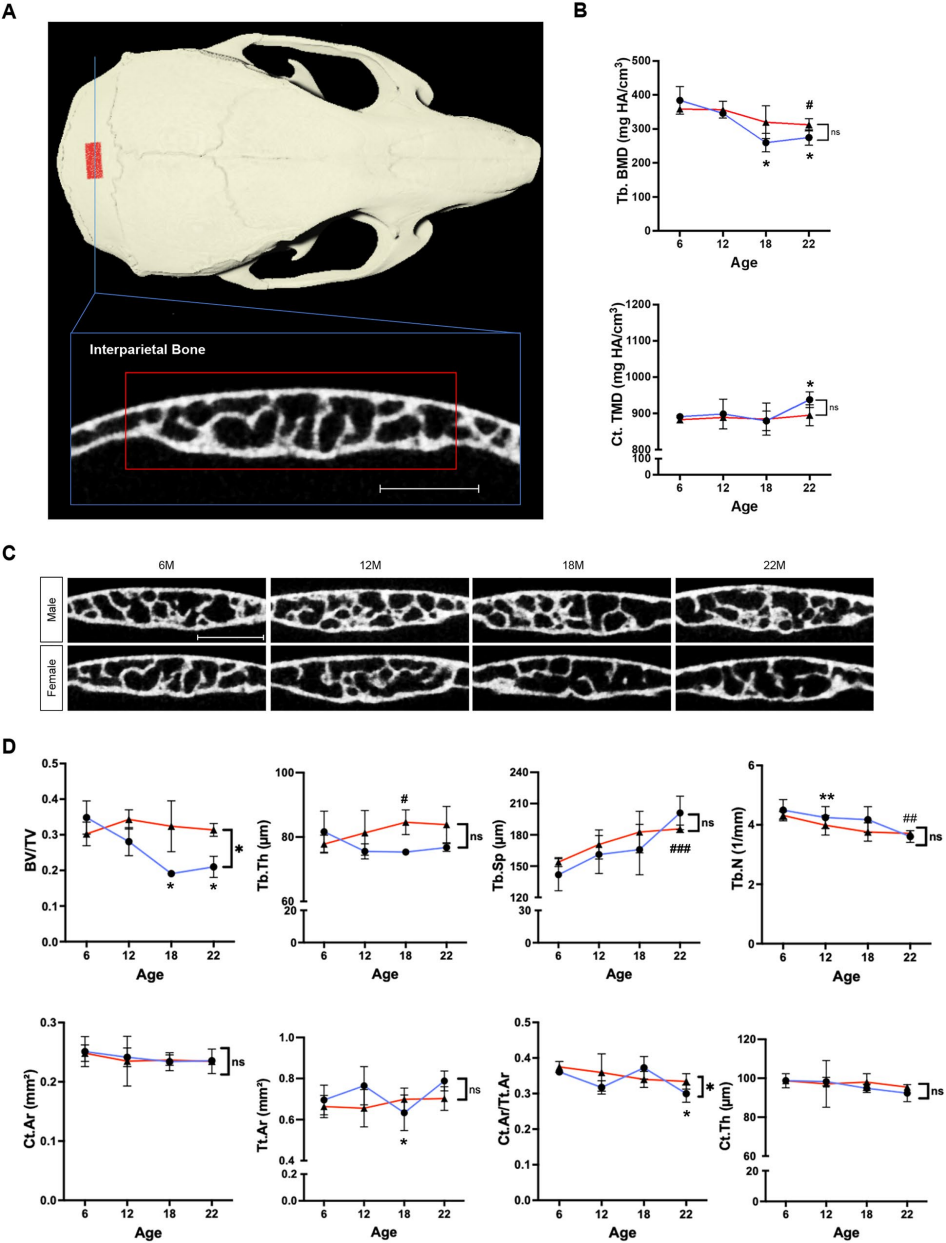
μCT imaging analysis of the interparietal bone. (**A**) 3D reconstruction of the skull and overview of VOI selection for the interparietal bone (red panel). Scale bar, 1 mm. (**B**) Interparietal trabecular bone mineral density as Tb.BMD in mg HA/cm^3^ and cortical bone mineral density as Ct.TMD in mg HA/cm^3^ for male (blue lines) and female (red lines) mice at 6, 12, 18, 22 months of age. (**C**) Original coronal cross-sectional images of the interparietal bone of male and female mice at 6, 12, 18, and 22 months of age. Scale bar, 1 mm. (**D**) Quantification of morphometric parameters of the interparietal bone of males (blue lines) and females (red lines). Trabecular bone volume fraction (BV/TV), trabecular number (Tb.N, 1/mm), trabecular thickness (Tb,Th, µm), trabecular separation (Tb.Sp, µm), cortical area (Ct.Ar, mm^2^), total area (Tt.Ar, mm^2^), cortical area fraction (Ct.Ar/Tt.Ar), and cortical thickness (Ct.Th, µm). n = 3 per group. *p < 0.05, **p < 0.01, ***p < 0.001 for males; ^#^*p* < 0.05, ^##^*p* < 0.01, ^###^*p* < 0.001 for females; ns, not significant.

To investigate the age-related changes in craniofacial bones formed through intramembranous ossification, we analyzed the interparietal bone (Fig. 5A). From 6 to 22 months of age, BV/TV decreased by 39.7% (*p* < 0.05) in males, Tb.Sp increased by 20.7% (*p* < 0.001) in females, Tb.N decreased by 14.1% (*p* < 0.01) in females, and Tb.BMD decreased by 28.4% (*p* < 0.05) in males and by 12.9% (*p* < 0.05) in females. For the interparietal cortical bone, Ct.Ar/Tt.Ar decreased by 17.0% (*p* < 0.05) in males, and Ct.TMD increased by 5.3% (*p* < 0.05) in males over time (Fig. 5B–D). These findings indicate that the changes in the interparietal bone were less pronounced for each measurement compared to those of the long bones.

**Figure 6:**
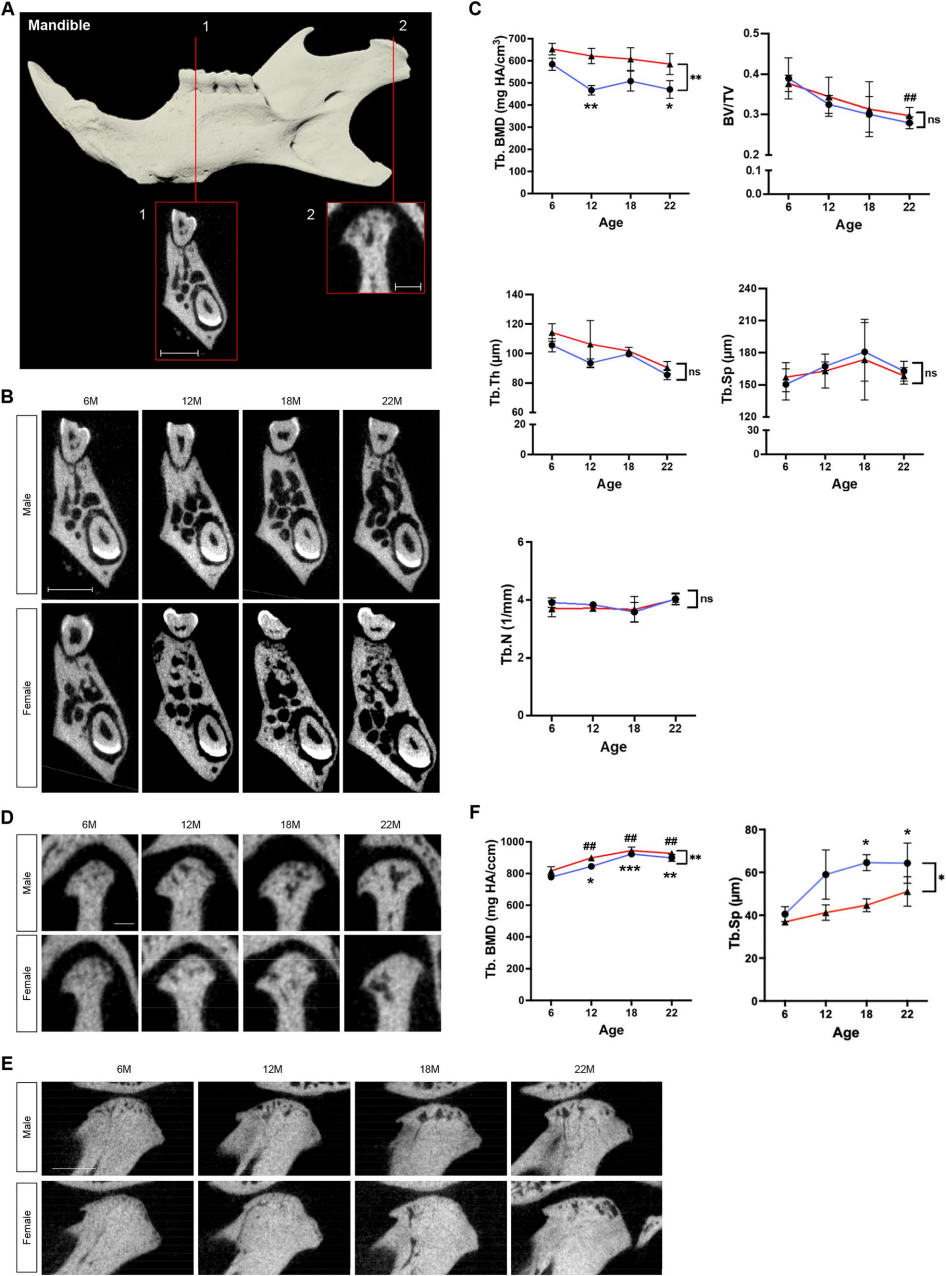
μCT imaging analysis for the mandible. (**A**) 3D reconstruction of the mandible (top) and overview of VOI selection for trabecular bone at M1 bifurcation (1) and the mandibular condyle (2). Scale bar 1 mm and 0.5 mm, respectively. (**B**) Original coronal cross-sectional images of mandibular trabecular bone at M1 bifurcation of male and female mice at 6, 12, 18, and 22 months of age. Scale bar, 1 mm. (**C**) Mandibular trabecular bone mineral density as Tb.BMD in mg HA/cm^3^ for male (blue lines) and female (red lines) mice at 6, 12, 18, 22 months of age. Quantification of morphometric parameters of mandibular trabecular bone of males (blue lines) and females (red lines). Trabecular bone volume fraction (BV/TV), trabecular thickness (Tb,Th, µm), trabecular space (Tb.Sp, μm), and trabecular number (Tb.N, 1/mm). (**D**) Original coronal cross-sectional images of mandibular condyle of male and female mice at 6, 12, 18, and 22 months of age. Scale bar, 0.5 mm. (**E**) Original sagittal cross-sectional images of mandibular condyle of male and female mice at 6, 12, 18, and 22 months of age. Scale bar, 0.5 mm. (**F**) Condylar trabecular bone mineral density as Tb.BMD in mg HA/cm^3^ for male (blue lines) and female (red lines) mice at 6, 12, 18, 22 months of age. Quantification of morphometric parameters of the mandibular condyle of males (blue lines) and females (red lines). Trabecular separation (Tb.Sp, µm). n = 3 per group. *p < 0.05, **p < 0.01, ***p < 0.001 for males; ^#^*p* < 0.05, ^##^*p* < 0.01, ^###^*p* < 0.001 for females; ns, not significant.

Finally, we analyzed the mandible, which is uniquely formed through both endochondral and intramembranous ossification^43^. To investigate the age-related effects on each type of ossification in the mandible, two separate regions were selected for analysis (Fig. 6A). First, the mandibular trabecular bone below the M1 bifurcation, which is formed by intramembranous ossification, was measured. From 6 to 22 months of age, BV/TV decreased by 21.0% (*p* < 0.01) in females, Tb.Th decreased by 19.1% (*p* < 0.05) in males and by 20.9% (*p* < 0.05) in females, and Tb.BMD decreased by 19.5% (*p* < 0.05) in males. There was no significant difference in other trabecular measurements in male and female mice (Fig. 6B,C). Next, the mandibular condyle, which forms by endochondral ossification, was measured. From 6 to 22 months of age, Tb.Sp increased by 58.8% (*p* < 0.05) in males, and Tb.BMD increased by 15.5% (*p* < 0.01) and by 13.4% (*p* < 0.01) in females. Interestingly, a greater change (Tb.Sp, *p* < 0.05; Tb.TMD, *p* < 0.01) was observed for male mice compared with female mice (Fig. 6D–F). A difference between the sexes was observed in the Tb.BMD of both the mandibular condyle and the mandibular body.

Our results from the μCT analyses were validated with histological staining (Fig. S1), which showed that the thickness and length of trabecular bones in the femur, tibiae, and vertebrae were decreased with aging in both male and female mice. Previous studies showed that the bone marrow in the femur and tibiae changes to yellow marrow, which is composed of adipocytes, with aging more drastically in female mice^44,45^. In agreement with these findings, the femoral and tibial bone marrow changed to yellow marrow in the femurs of female mice older than 18 months and in the tibia of female mice older than 12 months, whereas in male mice it remained as red marrow in these bones until the age of 22 months. In addition, as seen in the μCT images, there was increased invasion of blood vessels into the subchondral bone of the mandibular condyles, while the thickness of fibrocartilage on the condylar surface was intact. Thus, the change seen in the mandibular condyle differed from that observed in other bones.

## Discussion

Mice have been widely used in research on aging because of their small size and short lifespan, as well as conserved morphological changes and mechanisms compared to humans. Previous studies showed that C57BL/6J mice have an average lifespan of approximately 2 years^46,47^ and share features with humans, such as decline in ovarian follicles, irregular cycling and steroid hormone fluctuations, and irregular fertility^48–50^. In male and female mice (C57BL/6J, BALB/c, and C3H/He strains), the trabecular bone volume in long bones starts to decrease after approximately 6 months of age, with a greater reduction observed in females^38,39^. Furthermore, there is an increase in Tb.Sp and a decrease in Ct.Th with aging^38–40^, which are similar to the trends of bone loss seen in humans. One difference observed between humans and mice was the change in trabecular thickness, which decreases in humans with aging whereas it increases in mice^39,40^.

Reproductive hormones are a known factor for aging- and menopause-related bone loss^51,52^. In postmenopausal women, serum estradiol (E2) is drastically reduced while follicle-stimulating hormone (FSH) is increased^53–55^. Estrogen signaling is transduced by nuclear receptors ERα or ERβ. ERα signaling induces cell proliferation and differentiation of pre-osteoblasts in the cortical periosteum, which is required for cortical bone accrual and against bone resorption, although ERα signaling is not involved in the regulation of trabecular bone mass in mature osteoblasts and osteocytes^56,57^. By contrast, ERβ signaling represses bone formation in cortical bone, but not in trabecular bone^58^. Both ERα and ERβ signaling prevent cellular senescence in osteocytes due to p53 stabilization^59^. In osteoclasts, ERα signaling induces apoptosis, which prevents bone resorption in trabecular bones, but not in cortical bones^60,61^. Taken together, the balance between ERα and ERβ signaling may play a critical role in the regulation of bone growth and remodeling. Moreover, a deficit of estrogen affects bone formation and homeostasis; estrogen deficiency alters osteoblast differentiation^62^ and also dysregulates osteoclast differentiation^63–65^.

FSH levels are another factor related to menopausal and postmenopausal bone loss though osteoclast activation^66–68^. In an osteoarthritis mouse model, treatment with FSH accelerates inflammatory cytokine production and stimulates osteoclastogenesis, while treatment with E2 shows opposite effects^69^. Osteoporosis is also induced during androgen deprivation therapy in prostate cancers, as well with aging, in men^70,71^. Treatments with dihydrotestosterone and/or E2 improves suppressed cell viability due to oxidative stress in pre-osteoblasts, but not in mature osteoblasts^72^. In addition, androgen plays a role in the maintenance of trabecular bone mass through proliferation and mineralization of osteoblasts, but not of osteoclasts^57,73^. Based on the mouse life span, 3–6 months of age is referred as “matured adult”, 10–15 months of age is “middle-aged”, and 18–24 months of age is “old” (https://www.jax.org/research-and-faculty/research-labs/the-harrison-lab/gerontology/life-span-as-a-biomarker). In mice, the level of plasma testosterone is drastically reduced after 450 days (16 months) of age in males^74^. The level of serum luteinizing hormone (LH)^75^, neurons producing gonadotropin-releasing hormone (GnRH)^76^, and the amount of progesterone in the hypothalamus^77^ are significantly decreased in aged female C57BL/6J mice. In agreement with these findings, our results show that the quality and quantity of bones are altered at the middle-age, which is correlated with these hormonal changes.

Our findings indicate that the age-related changes in cortical and trabecular bone of the calvaria are less pronounced compared to those of long bones. One of the possible reasons for this observation is the different ossification mechanism involved in bone development: intramembranous ossification in craniofacial bones versus endochondral ossification in almost all bones in the trunk. Previous studies suggest that differences in the mode of ossification may lead to differences in cell proliferation, osteogenic differentiation, and in the lifespan of bone marrow stem cells^78^, as well as differences in the composition of the bone matrix^79,80^. Interestingly, calvarial trabecular bones show a higher bone turnover rate, with larger-size osteoclasts, compared with long bones and the mandible. By contrast, under E2 depletion conditions, bone turnover is upregulated in the calvaria and long bones, but not in the mandible^81^. Another possibility is the different mechanical forces and remodeling rate in craniofacial bones compared to long bones. It is well known that bone formation and resorption are strongly affected by mechanical forces^82–85^, and mechanical loading stress is converted to a series of biochemical reactions, which then activate osteoclasts and osteoblasts to promote bone remodeling^86^. Previous studies have revealed that lack of weight-bearing due to either prolonged spaceflight or bedrest leads to decreased bone mass^87,88^, while mechanical loading of the skeleton through various types of exercise results in increased bone mass^89,90^. Although there is no definitive study comparing mechanical forces between bones, the bones in the trunk would be more affected by body weight and gravity while the condyle in the temporomandibular joint would be more affected by occlusion. The mandibular and maxillary alveolar bone undergoes dramatic changes, which are accompanied by tooth loss. However, many risk factors contribute to the severity of alveolar bone loss with aging, such as hormone levels and individual dental health^91,92^. Periodontitis leads to significant loss of the alveolar bone and tends to be more severe in elderly women. However, laboratory mice are housed in sterile conditions, hence they are not affected by periodontitis. Therefore, the changes in the trabecular and cortical bone measurements observed for the mandibular alveolar bone reflect the effect of age and sex without the contribution of periodontitis.

When interpreting the measurements for mineral density, it is important to be aware of some of the limitations of μCT in terms of accuracy in bone and tissue mineral density. For instance, although μCT is a rapid and nondestructive method to evaluate bone mineralization compared to traditional methods such as microradiography, it is vulnerable to artifacts potentially due to scan parameters, beam hardening, partial-volume effects, photon starvation, photon scatter, and inappropriate sampling^28,93,94^. With that in mind, the mineral density measurements reported for trabecular bone were measured in BMD, which is calculated from average attenuation values of bone tissue and non-bone tissue from a VOI, whereas mineral density measurements reported for cortical bone were measured in TMD, which is calculated from bone tissue only. Trabecular BMD measurements correspond well with the trabecular morphometrics (i.e. bone volume and total volume ratio, trabecular separation and trabecular number); as the microarchitecture of trabecular bone deteriorates with aging, there is a gradual bone loss, which leads to a decrease in trabecular BMD values. One trabecular BMD measurement that did not follow this trend was the condylar trabecular BMD. Although there is an increase in trabecular spacing with aging, the total volume selected for analysis was significantly larger, which may have minimized the effect of non-bone tissue mineral density on the BMD measurement. Cortical TMD increases with aging, similar to what has been reported for previous studies^95,96^.

In summary, our measurements of age-related changes in trabecular and cortical bone of the femur, tibia, and vertebrae determined by μCT were consistent with previous findings. In this study, we found that craniofacial bones were differently and uniquely affected by age and sex.

## Supplementary Information

Supplementary Information.
