# Supplementary material for: Micro-computed tomography assessment of bone structure in aging mice
Source: Sci Rep. 2022 May 17;12:8117. doi: 10.1038/s41598-022-11965-4 (PMC9114112; doi:10.1038/s41598-022-11965-4)
Supplement: Supplementary file 1 — Supplementary Information. [file 41598_2022_11965_MOESM1_ESM.pdf]

Supplementary Information

Materials and Methods

Histology

Male and female C57BL/6J mice were purchased from The Jackson Laboratory at 26 weeks (6 months), 56 weeks (12 months), 72 weeks (18 months), and 90 weeks (22 months) of age (n = 3 per group). Mice were euthanized with carbon dioxide overdose followed by cervical dislocation at the above indicated time points. Mice were fixed with 4% paraformaldehyde in 0.1 M phosphate buffer (pH 7.4) through cardiac perfusion. The femur, tibia, vertebrae, and the skull were dissected and fixed with 4% paraformaldehyde (PFA) overnight. After decalcification with Immunocal decalcifier (StatLab, Texas, USA), Hematoxylin and Eosin (H&E) staining was performed on 5-μm paraffin sections of animals at 6 to 22 months of age (n = 3 per group), as previously described[1].

Reference

1 Iwata, J. *et al.* Modulation of noncanonical TGF-beta signaling prevents cleft palate in Tgfbr2 mutant mice. *The Journal of clinical investigation* **122**, 873-885, doi:10.1172/JCI61498 (2012).

Figure S1. Histological analysis of the femurs, tibiae (sagittal view), vertebrae (coronal view), and mandibular condyles (coronal view). H&E staining of the indicated bones of male and female mice at 6, 12, 18, and 22 months of age. Scale bars, 200 μm in the femur, tibia, and spine, and 100 μm in the mandibular condyle.

Figure S1

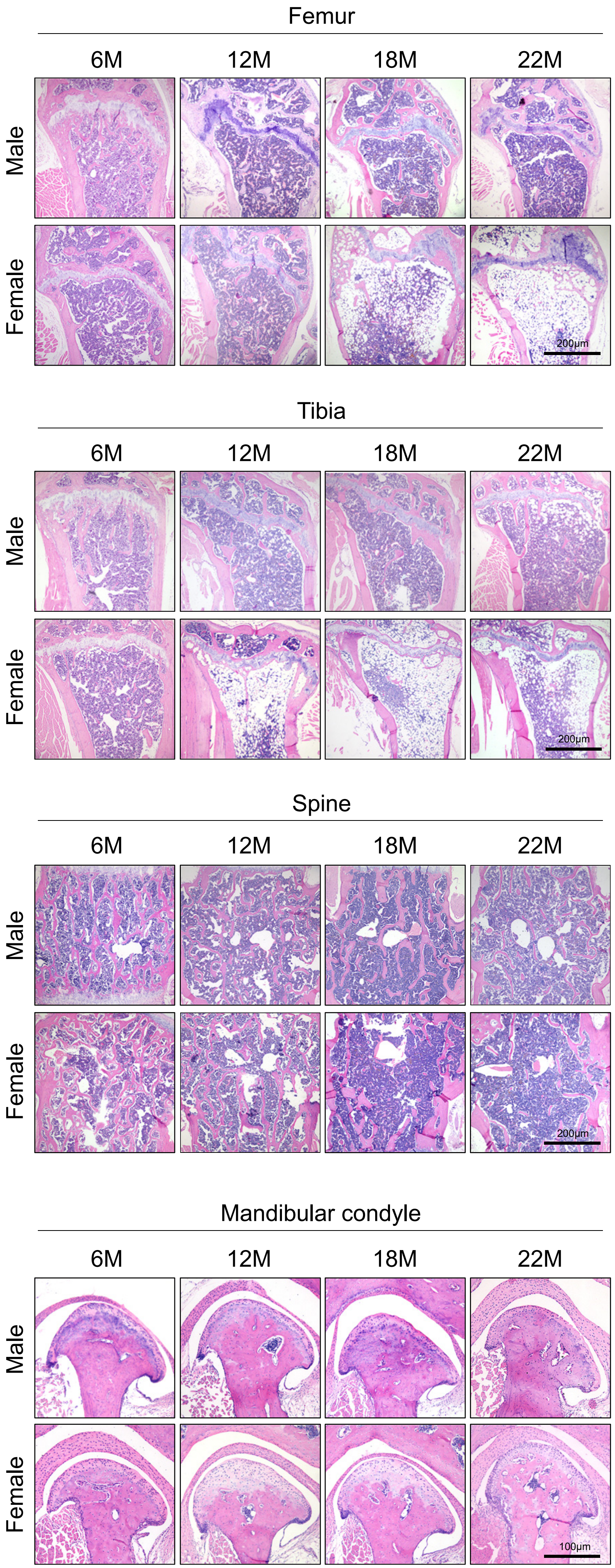
Femur
6M
12M
18M
22M
Male
Female
200μm
Tibia
6M
12M
18M
22M
Male
Female
200μm
Spine
6M
12M
18M
22M
Male
Female
200μm
Mandibular condyle
6M
12M
18M
22M
Male
Female
100μm
